# Influence of ultrasonic agitation on pH and antibiofilm activity of endodontic sealers – An *in vitro* study

**DOI:** 10.4317/jced.62477

**Published:** 2025-02-01

**Authors:** Francisca Livia Parente Viana, Clara Edvirgens Oliveira Sousa, Ana Grasiela Limoeiro, Murilo Priori Alcalde, Rodrigo Ricci Vivan, Marco Antônio Hungaro Duarte, Ericka Tavares Pinheiro, Bruno Carvalho Vasconcelos

**Affiliations:** 1 Post-graduate Program in Dentistry, Faculty of Pharmacy, Dentistry and Nursing, Federal University of Ceará, Fortaleza, CE, Brazil; 2 Sobral Dental School, Federal University of Ceará – Sobral Campus, Brazil; 3 Department of Dentistry, Endodontics and Dental Materials, Bauru Dental School, University of São Paulo, Bauru, SP, Brazil; 4 Department of Dentistry, São Paulo School of Dentistry, University of São Paulo, São Paulo, Brazil

## Abstract

**Background:**

The influence of ultrasonic agitation (UA) on the pH and antibiofilm activity of AH Plus Jet (AHP) and BioRoot RCS (BCS) sealers was evaluated. Subgroups were created based on the sealer/UA application.

**Material and Methods:**

The pH was measured at 30 min., 3, 24, 72, 168 h. Antibiofilm activity was determined by direct and indirect contact tests (DCT/ICT) on dual-species biofilms (Enterococcus faecalis and Streptococcus oralis). Bacterial survival was assessed by colony-forming unit (CFU) counting. The Mann-Whitney test was applied for th pH analysis whereas the Kruskall-Wallis and Dunn tests were employed for the DCT/ICT evaluations.

**Results:**

BCS presented the highest pH at all time points (*p*<0.05). Related to UA, it significantly reduced the pH at 30 min., 3, 24 and 72 h (*p*<0.05). In the DCT, a significant reduction in CFUs was observed in the BCS and BCS/UA groups compared to the control and AHP/UA group. BCS also showed the best results in the ICT (*p*<0.05).

**Conclusions:**

It was concluded that UA reduced pH and did not improve the sealers’ antibiofilm activity. BCS showed the highest pH values and antibiofilm activity.

** Key words:**Antibiofilm activity, Enterococcus faecalis, Streptococcus oralis, Root canal sealers, Ultrasonic activation.

## Introduction

Bacterial biofilms are the main etiological factors in periapical diseases (1,2). The removal of intraradicular biofilms during treatment and the prevention of bacterial recolonization are critical factors for the success of endodontic treatment. However, even after chemo-mechanical preparation and the use of intracanal medication, complete elimination of microorganisms does not occur (1). Therefore, the primary objective of root canal obturation is to ensure a thorough and long-lasting sealing after root canal disinfection (3). Additionally, employing endodontic sealers with strong antimicrobial properties is an important consideration (1), as they may act against residual biofilms present in hard-to-reach areas of the root canals.

Various endodontic sealers have been developed to achieve these objectives. AH Plus (Dentsply/De Trey GmbH, Konstanz, Germany), an epoxy-amine resin-based sealer, is one of the most widely used and scientifically tested endodontic sealers. It is considered by many as the gold standard among endodontic sealers, standing out for its excellent physicochemical properties (4) and antimicrobial activity (1,5), combined with appropriate biological properties (5). However, its antiseptic capacity is limited after setting (6).

Calcium silicate-based sealers are favoured for their bioactivity and biocompatibility (7). A key feature is their capacity to alkalize the environment and release calcium ions, contributing to their osteogenic potential, biocompatibility, and antibacterial properties (8). BioRoot RCS (Septodont, Saint Maur-des-Fosses, France), a tricalcium silicate-based sealer available in powder/liquid form, is known for its excellent adhesion (9), high fluidity and radiopacity (10). Additionally, it is biocompatible (7), promotes hard tissue deposition (10), and possesses antimicrobial efficacy (11).

The development and evolution of endodontic sealing materials are accompanied by ongoing research into new protocols. In this context, ultrasonic agitation of endodontic sealers has been proposed to improve the quality of root canal obturation. The literature describes the effect of ultrasonic agitation in adapting endodontic sealers during root canal obturation. It promotes greater penetration of sealers into dentinal tubules and reduced gap areas along the canal circumference (12-14). Moreover, studies have shown that the ultrasonic agitation can accelerate the setting reaction and improve the mechanical properties of sealers (12-14).

Although ultrasonic agitation may enhance the physicochemical properties and intra-tubular penetration of endodontic sealers, there is limited information regarding its impact on the antimicrobial activity of these materials. Alcalde *et al*. (15) reported that ultrasonic activation improved the antimicrobial efficacy of AH Plus against *Enterococcus faecalis* within dentine tubules. However, there is currently no data available on the antimicrobial activity of calcium silicate-based sealers following ultrasonic agitation. Therefore, this study aims to evaluate the influence of ultrasonic agitation on the activity of AH Plus Jet, a resin-based sealer, and BioRoot RCS, a bioceramic sealer, against dual-species biofilms. Additionally, the pH of the sealers will be measured before and after ultrasonic agitation. The null hypothesis being tested is that ultrasonic agitation does not result in changes in pH or enhance the antibiofilm activity of endodontic sealers.

## Material and Methods

**Figure 1:**
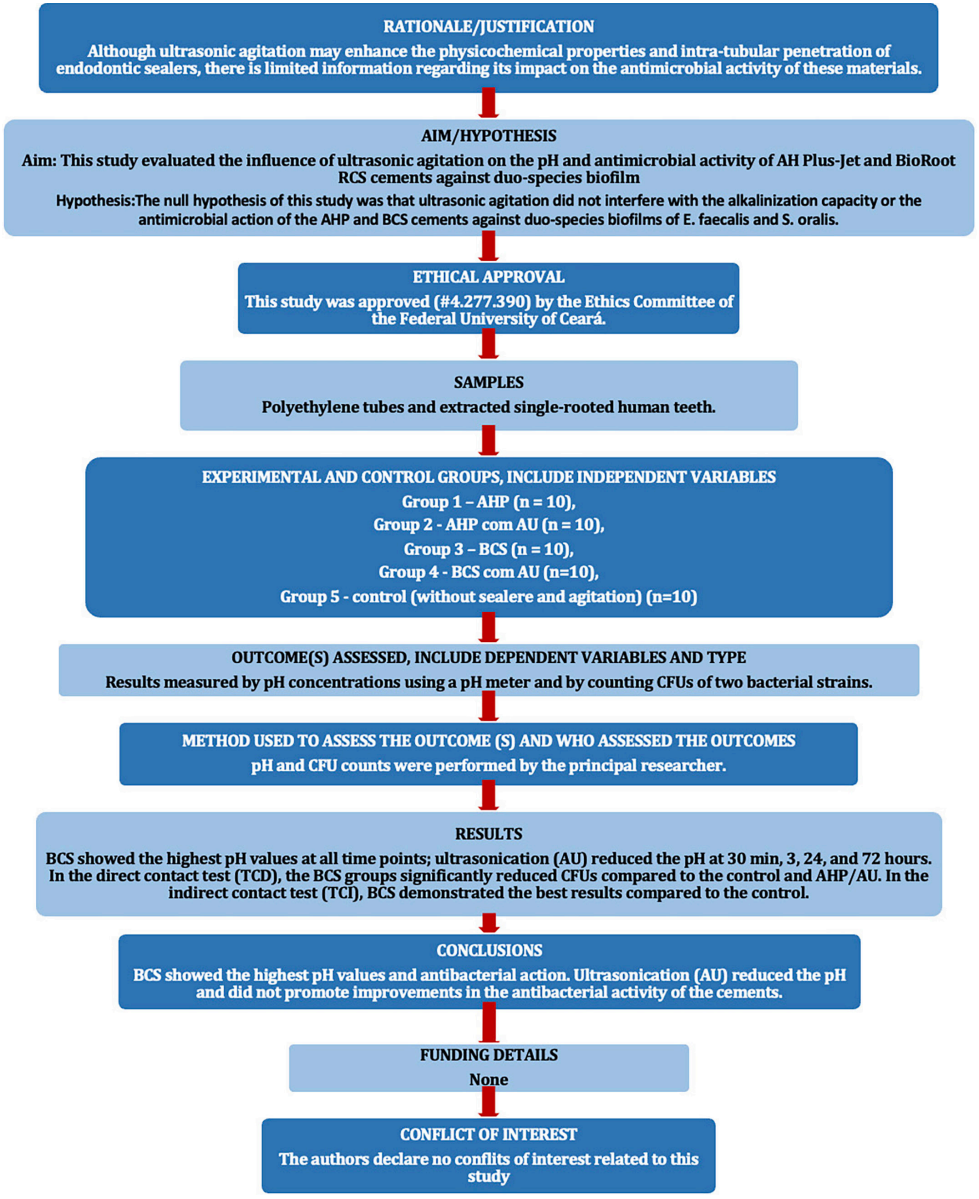
PRILE flowchart.

The PRILE 2021 guidelines (Fig. 1) were used to plan and report the present laboratory study. The endodontic sealers used were AH Plus Jet (AHP; Dentsply/De Trey GmbH Konstanz, Germany) and BioRoot RCS (BCS; Septodont, Saint Maur-des-Fosses, France), whose compositions are presented in  Table 1. Regardless of the analysis, experimental groups were divided based on the sealer used and the application or not of ultrasonic agitation (UA).

-pH determination 

The procedures for this test followed those described by Silva *et al*. (12). G*Power v3.1 for Mac (Heinrich Heine University Düsseldorf) was used for sample size calculation, selecting the T-test. Data from a previous study were utilized, with the effect size for the present study established at 1.54. An alpha error of 0.05, a beta power of 0.95, and an N2/N1 ratio of 1 were also stipulated. A total of 8 samples per group was indicated as the ideal size necessary to observe significant differences. Due to the risk of sample loss, an additional 20% was employed, resulting in 10 specimens per group.

The specimens (n = 10) for this assay were obtained by inserting the sealers into tubes using Paiva-type pluggers until filled. In the groups subjected to ultrasonic agitation (UA), a smooth conical insert (E1; Helse Ultrasonics, Santa Rosa do Viterbo, SP, Brazil) coupled to a piezoelectric ultrasonic device (Ultrawave Piezo Ultrasonic Scaler; Ultradent Products Inc., South Jordan, UT, USA) programmed at power #3 (approximately 30%), was introduced into the center of the mass of the material and subsequently activated. Two cycles of 20 seconds of UA were performed in different directions (vertical and horizontal); afterward, vertical condensation was performed again, and no additional filling was required.

After filling, each specimen was individually immersed in a sterile and DNAse and RNAse-free Falcon tube (Techno Plastic Products Ag., Trasadingen, Switzerland) containing 10 mL of deionized water and kept in an incubator at 37°C and 100% humidity throughout the experimental period; the original pH of the water was determined to be 7.1. The alkalinizing potential was determined at 30 minutes, 3 hours, 24 hours, 72 hours, and 168 hours, during which the specimens were carefully placed in a new Falcon tube with the same volume of fresh deionized water for each period. The pH was determined using a previously calibrated pH meter with solutions of known pH (4, 7, and 10).

-Antimicrobial effectiveness of sealers on dual-species biofilms-direct and indirect contact tests

Human-extracted teeth were used; this analysis was previously reviewed and approved by the local Research Ethics Committee (#4.277.390). Sample size calculation was performed using G*Power v3.1 for Mac (Heinrich Heine, Universität Düsseldorf), and the t-test was selected. Data from a previous study that evaluated antibiofilm activity on biofilms formed on human dentin blocks (16) were used, and the effect size in the present study was set (=1.54). An alpha error of 0.05, a beta power of 0.95, and an N2/N1 ratio of 1 were also established. A total of 8 samples per group was indicated as the ideal sample size necessary to observe significant differences. Due to the risk of sample loss, 20% more samples were included, resulting in 10 specimens per group.

Gram-positive bacterial strains of *Enterococcus faecalis* (ATCC 29212) and *Streptococcus oralis* (PB 182) were used. The bacterial stocks were preserved in Brain Heart Infusion (BHI; Kasvi, São José dos Pinhais, PR, Brazil) medium containing 20% glycerol in a freezer at -80ºC. The bacterial strains were reactivated by inoculating 200 μL into tubes containing 9 mL of BHI broth and 1 mL of 10% glucose in duplicate and incubating at 37°C for 18 hours. The culture purity was verified by Gram staining, and the inoculum was standardized to an optical density of 0.5 (620 nm) for turbidity equivalent to a growth of 1-2 x 108 CFU/mL.

Human teeth with fully formed roots were used as a substrate for biofilm growth (17). The roots were sectioned into blocks measuring 4.0 mm x 4.0 mm x 2.0 mm [width x length x thickness] using a 0.3 mm thick diamond disc in a cutting machine at 200 rpm under abundant irrigation. The resulting blocks were immersed in 17% EDTA (Dentsply-De Trey, Konstanz, Germany) for 3 minutes to remove dentin debris and then placed in a test tube containing distilled water and sterilized in an autoclave at 121°C, 1 ATM, for 15 minutes.

Microbiological procedures and handling of the sterilized dentin blocks were performed in a laminar flow chamber. After the overnight growth of the two species (*E. faecalis* and *S. oralis*), the culture purity was verified, and the bacterial inoculum was standardized to a 0.5 density on the McFarland scale. The dentin blocks were placed in the wells of 24-well culture plates, where each well received 100 μL of *E. faecalis*, 100 μL of *S. oralis*, 700 µL of sterile BHI broth, and 100 μL of 10% sucrose. The culture plates with the submerged dentin blocks were incubated at 37°C and 5% CO2 for 21 days for biofilm formation. Every 48 hours, the BHI medium of each sample was replaced without adding new microorganisms.

For the direct contact material/biofilm test, 0.05 mL of fresh sealer was placed on the biofilm formed on the dentin blocks. In the indirect contact test, a sterile nitrocellulose membrane (Merck Millipore Ltd, Tullagreen, Cork, Ireland) with 0.22 μm pores was placed on the biofilm before applying the sealer, which is a membrane-restricted test. The indirect contact test was used to evaluate the ability of antimicrobial components of the sealers to penetrate a physical barrier. For the groups subjected to UA, the sealer was inserted into a sterile syringe, followed by ultrasonic agitation, and then placed on the biofilm-covered block; ten dentin blocks were allocated for each group (n = 10). The dentin block/sealer samples were positioned in cell culture plates, and the contact time was maintained for 24 hours at 37°C and 5% CO2, regardless of the contact pattern.

After the contact period, the sealer was removed from the surface of the dentin block in the direct contact test. In the indirect contact test, the cellulose membrane with the sealer was discarded. Ten dentin blocks with biofilms without contact with the materials were used as controls. After sealer removal, the dentin blocks were individually transferred to a vial containing 2 mL of sterile saline solution and gently agitated to remove loosely adhered cells. They were then transferred to another vial containing 2 mL of saline solution and vortexed for 1 minute, alternating with an ice bath. A serial dilution was performed in saline solution, and 10 μL aliquots from each dilution were plated on BHI agar and incubated for 48 hours at 37°C and 5% CO2 to count the total bacterial load of both species and on M-Enterococcus agar to allow the growth of *E. faecalis* colonies only. Colony-forming units (CFUs) were counted after 48 hours of incubation at 37°C and 5% CO2. The bacterial count of *S. oralis* was calculated by subtracting the bacterial count on M-Enterococcus agar from the total bacterial count.

-Statistical Analysis 

Data were tabulated and subjected to the Shapiro-Wilks test to verify normality. The Mann-Whitney test was used to analyze pH, the two-factor analysis of the sealers, and ultrasonic agitation, while the Kruskal-Wallis and Dunn tests were used for the direct and indirect contact tests. Significance was set at 5.0%.

## Results

The BCS sealer recorded the highest pH values, demonstrating a significant difference from the AHP at all evaluated time points, irrespective of ultrasonic agitation (*P* < 0.05) ( Table 2). Ultrasonic agitation notably decreased the pH levels of both AHP (at 30 minutes, 24 hours, and 72 hours) and BCS (at 3, 24, and 72 hours) (*P* < 0.05).

Both BCS groups demonstrated a significant reduction in total bacterial count compared to the AHP/UA and control groups (all *P* < 0.05) in the direct contact test ( Table 3). More specifically, the count of E. faecalis indicated that the BCS groups, with and without UA, showed greater antibacterial activity than the control group (*P* < 0.05). Similarly, the BCS groups differed significantly from the AHP groups, regardless of UA (*P* < 0.05). In turn, the groups had no significant differences in S. oralis growth ( Table 3).

The indirect contact test showed that BCS, regardless of UA, was significantly more effective than the control (*P* < 0.05) in reducing total bacteria and *E. faecalis* counts ( Table 4). The BCS/UA group also showed a notable reduction in *E. faecalis* compared to AHP without UA (*P* < 0.05). In contrast, no significant differences were found in *S. oralis* counts.

A two-factor analysis revealed that BCS significantly outperformed AHP (*P* < 0.0001) in reducing total bacterial count and *E. faecalis* in both tests. However, ultrasonic agitation did not enhance the antibacterial effectiveness of the sealers.

## Discussion

The present study examined the impact of ultrasonic agitation on endodontic sealers’ pH and antimicrobial activity. To the best of the authors’ knowledge, this is the first investigation into the effect of ultrasonic agitation on the antimicrobial properties of BioRoot RCS sealer. The UA reduced the pH of the sealer and did not affect the antimicrobial action of the sealers, so the null hypothesis was rejected.

A notable strength of this study is the use of a mature biofilm model (21 days of growth) on human dentin, as well as a variety of strategies to assess the activity of the endodontic sealers against biofilms. The quantitative direct contact test effectively simulates the interaction between biofilms and sealers, allowing for the measurement of bacterial growth after treatment (1). Conversely, the indirect contact test mimics the limited access of the sealers to biofilms, evaluating the diffusion capacity of their antimicrobial components in the presence of a physical barrier (1). A limitation of this study is the association of only two bacterial species, which do not fully represent the nature of residual infections after endodontic procedures. However, the dual-species biofilm model could offer advantages over a monospecies biofilm, as *E. faecalis* is known to form a dense biofilm structure on dentin alongside Streptococcus, effectively penetrating dentinal tubules (18).

Ultrasonic agitation did not elevate the pH levels of calcium silicate-based sealers. In fact, it resulted in reduced pH for both sealers examined, aligning with the results reported by Kim *et al*. (19). This outcome contrasts with earlier studies suggesting that ultrasonic activation could potentially raise the pH levels of calcium silicate-based sealers. The observed results may be attributed to the heating effect on the sealer, which not only shortens the setting time (19) but also reduces solubility (20), thereby limiting the ionic dissociation of its components. When AH Plus is subjected to heat, the setting time decreases due to the accelerated reaction of amine groups, which are crucial for polymerization in epoxy resin-based sealers (5). Similarly, BioRoot RCS sealer exhibits a notably reduced setting time in heated conditions, likely due to the enhanced activity of calcium chloride, a setting accelerator, at elevated temperatures (5,19). Furthermore, its solubility diminishes when heated (19).

In line with the findings of pH analysis, ultrasonic agitation did not improve the antibacterial activity of the sealers. Our findings contrast with a previous study (15), which indicated that ultrasonic activation of AH Plus enhanced its effectiveness against *E. faecalis* within dentinal tubules. The differences between the studies may arise from the biofilm models used to evaluate the antimicrobial efficacy of the sealers. For instance, the mature biofilms (21 days) of two species used in our study are likely to exhibit greater resistance than younger biofilms of a single species analysed in the earlier study (15). However, more research is needed to better understand how ultrasonic agitation affects the antimicrobial activity of sealers, as current studies are limited and use different microbiological methods.

As expected, BioRoot RCS exhibited higher pH values than AH Plus (20-22). It also had the most effective activity against dual-species biofilms in the direct contact test, consistent with earlier research (23). Its antimicrobial properties can be attributed to its ability to maintain elevated alkalinity levels over prolonged periods, as indicated by previous studies (16,24). When in contact with water, calcium silicate-based sealers produce a calcium silicate hydrate gel (CSH, CaO SiO H2O), leading to the formation of calcium hydroxide. The dissociation of Ca(OH)2 releases calcium (Ca2+) and hydroxyl (OH-) ions, which in turn raises the pH and inhibits bacterial viability (24). A significant correlation has also been observed between the release of free Ca2+ and silicon (Si4+) ions and the antibacterial effects of bioceramic sealers (8). These released ions may contribute to bacterial membrane depolarization and cell lysis (8).

In the indirect contact test, the results once again favoured BioRoot RCS. A barrier restricting direct contact between materials and the biofilm indicates a requirement for soluble agents. These agents should be able to navigate through the moisture in both the material and the biofilm (1). Consequently, our findings can be attributed to the diffusion of ions from silicate-based sealers, emphasizing the importance of enhanced solubility and diffusion capacity in the antimicrobial effectiveness of these materials. From the clinical point of view, the solubility of silicate-based sealers may affect their efficacy within the dentinal tubules, allowing them to reach microorganisms located in less accessible areas. Conversely, AH Plus Jet exhibited limited efficacy against biofilms, aligning with prior findings (5). The neutral to mildly alkaline pH and low solubility of AH Plus (25) may have limited the effectiveness of its components against biofilms in direct and indirect contact tests.

## Conclusions

Considering the limitations of the current study, it can be concluded that the ultrasonic activation was found to lower the pH of the evaluated sealers without enhancing their antibacterial effectiveness. Additionally, the bioceramic sealer BioRoot RCS demonstrated the highest pH values at the assessed time points, along with pronounced antibacterial activity against the dual-species biofilm of *E. faecalis* and *S. oralis* in both direct and indirect contact tests.

## Figures and Tables

**Table 1:** Composition of sealers.

	Component A / Powder	Component A / Liquid
AH Plus Jet (Dentsply / De Trey GmbH)	Bisphenol-A Epoxy Resin (25-50%)	N,N′-dibenzil-5-oxanonadiamine-1,9 (10-25%)
	Bisphenol-F Epoxy Resin (2,5-10%)	Aminoadamantano (2,5-10%)
	Calcium Tungstate	Calcium Tungstate
	Zirconium Oxide	Zirconium Oxide
	Sílica	Sílica
	Iron Oxide Pigments	Silicone Oil
BioRoot RCS	Tricalcium Silicate (25-50%)	Calcium Chloride Dihydrate
(Septodont, Saint Maur-des-Fosses, France)	Zirconium Oxide (25-50%)	Polycarboxylate
	Povidone	Purified Water

**Table 2:** Means (minimum and maximum values) of pH found at different periods.

Sealers	30 min.		3 hrs		24 hrs		72 hrs		168 hrs	
	Means (min. - max.)		Means (min. - max.)		Means (min. - max.)		Means (min. - max.)		Means (min. - max.)	
	With AU	Without AU	With AU	Without AU	With AU	Without AU	With AU	Without AU	With AU	Without AU
AH Plus Jet	8.62 ^b,A^	7.55^b,B^	7.9^b,A^	8.09^b,A^	8.54^b,A^	7.76^b,B^	7.62^b,A^	7.05^b,B^	7.08^b,A^	7.52^b,A^
	(8.4 – 9.3)	(7.1 – 8.2)	(7.5 – 8.6)	(7.6 – 8.7)	(8.1 – 8.9)	(7.4 – 9.0)	(6.5 – 9.1)	(6.7 – 8.8)	(6.0 – 9.4)	(7.1 – 8.9)
BioRoot RCS	10.44 ^a,A^	10.1^a,A^	9.86^a,A^	9.27^a,B^	10.0^a,A^	9.71^a,B^	9.64^a,A^	8.57^a,B^	8.4^a,A^	8.75^a,A^
	(9.9 – 10.9)	(9.3 – 10.9)	(9.4 – 10.6)	(9.0 – 9.9)	(9.6 – 10.3)	(9.2 – 10.1)	(8.3 – 10.4)	(7.6 – 10.1)	(7.1 – 9.8)	(7.7 – 10.3)

a,b Lowercase letters in superscript indicate significant differences between the groups in the evaluated periods according to Mann-Whitney tests (*p*< 0.05). A,B Uppercase letters in superscript indicate significant differences according to Mann-Whitney tests (*p*< 0.05) between the same cement with or without ultrasonic agitation.

**Table 3:** Median (min – max) of bacterial count (log 10) from the direct contact test against the duo-species biofilm of E. faecalis and S. oralis.

	AHP	AHP/AU	BCS	BCS/AU	CONTROL
	Median (Min-Max)	Median (Min-Max)	Median (Min-Max)	Median (Min-Max)	Median (Min-Max)
TOTAL	4,84 ^abc^^, B^	4,57 ^bc^^, B^	0,93 ^a^^, A^	1,69 ^a^^, A^	6,84 ^c^^, B^
	(0,00 – 6,31)	(4,14 – 6,03)	(0,00 – 4,19)	(0,00 – 3,23)	(5,43 – 7,40)
ENTEROCOCCUS FAECALIS	4,80 ^bc^^, B^	4,46 ^bc^^, AB^	0,00 ^a^^, A^	1,69 ^ab^^, A^	6,43 ^c^^, B^
	(0,00 – 6,25)	(4,23 – 5,95)	(0,00 – 4,08)	(0,00 – 3,11)	(5,09 – 7,48)
STREPTOCOCCUS ORALIS	0,00 ^a, A^	3,71 ^a^^, A^	1,93 ^a^^, A^	1,30 ^a^^, A^	5,17 ^a^^, A^
	(0,00 – 5,34)	(0,00 – 5,25)	(0,00 – 3,50)	(0,00 – 2,75)	(0,00 – 6,72)

A,B Different superscript letters indicate significant differences between the same treatment/cement according to the Kruskal-Wallis tests and Dunn’s comparison test (*p*<0.05). a,b Different superscript letters indicate significant differences between the groups according to the Kruskal-Wallis tests and Dunn’s comparison test (*p*<0.05).

**Table 4:** Median (min – max) of bacterial count (log 10) from the indirect contact test against the duo-species biofilm of E. faecalis and S. oralis.

	AHP	AHP/AU	BCS	BCS/AU	CONTROL
	Median (Min-Max)	Median (Min-Max)	Median (Min-Max)	Median (Min-Max)	Median (Min-Max)
TOTAL	5,30 ^ab, B^	5,32 ^ab, B^	4,88 ^a, A^	4,07 ^a, A^	6,13 ^b, B^
	(3,75 – 5,91)	(4,57 – 5,64)	(2,39 – 5,20)	(3,17 – 5,13)	(5,17 – 6,75)
ENTEROCOCCUS FAECALIS	5,33 ^bc, B^	5,11^ abc, B^	4,57 ^ab, A^	4,017^ a, A^	6,17 ^c, B^
	(3,39 – 6,11)	(4,53 – 5,62)	(0,00 – 5,11)	(3,17 – 4,86)	(5,00 – 6,74)
STREPTOCOCCUS ORALIS	3,15 ^a, A^	4,32 ^a, A^	4,25 ^a, A^	3,39 ^a, A^	5,03 ^a, A^
	(0,00 – 5,54)	(0,00 – 4,91)	(0,00 – 4,60)	(0,00 – 4,79)	(0,00 – 5,47)

A,B Different superscript letters indicate significant differences between the same treatment/cement according to the Kruskal-Wallis tests and Dunn’s comparison test (*p*<0.05). a,b Different superscript letters indicate significant differences between the groups according to the Kruskal-Wallis tests and Dunn’s comparison test (*p*<0.05).

## Data Availability

The datasets used and/or analyzed during the current study are available from the corresponding author.
